# The Weight of Eloquence in Motor Area Glioblastoma: Oncologic Outcome After nTMS-Guided Surgical Resection

**DOI:** 10.3390/neurosci6040124

**Published:** 2025-12-03

**Authors:** Luca Sartori, Samuel Luciano Caliri, Roberto Colasanti, Pietro Dalla Zuanna, Nicola Bresolin, Valentina Baro, Pietro Ciccarino, Francesco Volpin, Franco Chioffi, Luca Denaro, Andrea Landi

**Affiliations:** 1 Academic Neurosurgery, Department of Neuroscience, University of Padova, 35128 Padova, Italy; 2 Department of Neurosurgery, Padova University-Hospital, 35128 Padova, Italy

**Keywords:** glioblastoma, motor system, navigated transcranial magnetic stimulation, nTMS, motor mapping, eloquent area, functional preservation, survival outcome

## Abstract

Background: Glioblastomas (GBMs) in eloquent areas, particularly within the motor system, represent a significant surgical challenge due to the risk of postoperative neurological deficits. This study evaluates the effectiveness of a structured preoperative protocol, including nTMS-guided motor mapping, to optimize surgical outcomes and minimize neurological deficits, with a particular focus on the timing of adjuvant oncological therapy initiation. Methods: A retrospective analysis was conducted on 44 GBM patients, divided into two groups: 11 with motor area lesions (group A) and 33 with non-eloquent lesions (group B). All patients underwent a standardized preoperative protocol. Surgical outcomes (EORs), neurological function (MRC score and KPS index), time to oncological therapy initiation and survival (OS and PFS) were compared between groups. Results: Both groups achieved high rates of GTR without significant differences in EOR (72.7% group A vs. 78.8% group B). Although group A exhibited a higher incidence of postoperative motor deficits, motor function at three-month follow-up was similar between groups. Time to initiation of oncological therapy did not differ between groups (40.6 days group A vs. 41.9 days group B, *p* = 0.719), highlighting that preservation of motor function helped minimize delays in starting oncological therapy. No significant differences were found in survival outcomes. Conclusions: A structured preoperative protocol incorporating nTMS motor mapping allows for safe and aggressive resection of motor-area GBMs. This approach effectively mitigates the risk of delays in initiating adjuvant oncological therapy, optimizing the patient prognosis. Further studies are needed to explore the long-term benefits of this protocol in both functional and oncological outcomes.

## 1. Introduction

Glioblastoma (GBM) remains a highly aggressive primary brain tumor, characterized by significant neurological morbidity and dismal survival despite multimodal treatment strategies [1,2,3]. Due to its intrinsic infiltrative nature, complete resection is unattainable, as tumor cells invariably invade surrounding brain tissue. Even historically radical approaches, such as hemispherectomy, have failed to demonstrate a survival benefit [4].

Nevertheless, surgical resection remains a cornerstone of GBM treatment, primarily because it influences two well-established prognostic factors: extent of resection (EOR) and residual tumor volume (RV). Numerous studies have demonstrated that a higher EOR and minimal RV correlate with prolonged overall survival (OS), leading to an increasingly aggressive surgical philosophy [5,6,7,8]. This evidence has justified the pursuit of maximal resection strategies, including supratotal resection or “flairectomy,” when feasible [9,10].

However, the potential oncologic benefits of radical resection must be carefully balanced against the imperative of functional preservation. In addition to EOR and RV, postoperative neurological function has been recognized as an independent prognostic factor [11,12,13,14]. Recent evidence suggests that newly acquired neurological deficits may negatively impact OS, regardless of whether gross total resection (GTR) is achieved [15,16,17]. While the precise mechanisms underlying this association remain unclear, postoperative neurological deterioration likely affects quality of life, delays adjuvant therapy, and contributes to an overall worse prognosis. Consequently, neurosurgeons are faced with a persistent dilemma: should they prioritize oncologic outcomes by maximizing resection, even at the risk of functional impairment, or should they prioritize neurological preservation, potentially at the cost of reduced tumor control?

This question becomes even more critical in cases of eloquent-area glioblastomas, particularly those located within or adjacent to the motor system [18,19]. Motor-area GBMs constitute a distinct and highly infiltrative subgroup of gliomas involving the primary motor cortex (M1), premotor areas, or the corticospinal tract (CST). Their location inherently increases the risk of postoperative neurological deficits, which in turn may compromise functional independence and delay the initiation of adjuvant therapy. While extensive resection in these cases is often more challenging, the actual impact of tumor eloquence on survival remains poorly understood due to the limited literature on the topic. However, clinical experience clearly suggests that motor-area GBMs are associated with lower rates of extensive resection and a higher incidence of functional decline, both of which could negatively influence prognosis.

It is reasonable to hypothesize that glioblastomas located within the motor system inherently confer a poorer prognosis compared to non-eloquent GBMs, solely due to their location. The combination of reduced EOR, increased RV, and a higher incidence of postoperative deficits may contribute to this survival disadvantage.

In recent years, several technological advancements have been introduced to mitigate these risks and enhance the safety of glioblastoma surgery [20,21,22,23,24]. Neuronavigation, fluorescence-guided resection, intraoperative neurophysiological monitoring and direct cortical and subcortical mapping have significantly improved surgical precision and functional outcomes [25,26,27,28,29]. More recently, navigated transcranial magnetic stimulation (nTMS) has emerged as a valuable preoperative tool for motor mapping, allowing for a more individualized surgical strategy [30,31,32,33,34]. Preliminary evidence suggests that nTMS may improve EOR in motor-area GBMs compared to intraoperative mapping alone [31,34], yet its ability to fully equalize surgical risks between eloquent and non-eloquent tumors remains unproven.

In this study, we present a retrospective, single-center analysis of patients with high-risk motor-area GBMs, aiming to evaluate the efficacy of our surgical protocol, in which nTMS represents the first step in a structured decision-making process. Specifically, we investigate whether nTMS-guided planning can mitigate the surgical risks associated with eloquent tumor location and influence oncologic outcomes.

## 2. Materials and Methods

### 2.1. Patient Selection

Since March 2020, the patients with lesions in eloquent areas underwent a specific planning protocol at the Department of Neurosurgery and were prospectively entered into a database available for retrospective analysis. Subsequently, we collected data from all the patients affected by motor-area GBMs and operated between January 2022 and December 2023. They were classified as high-risk motor-area GBMs according to the risk stratification model presented in the literature which asks for the distance between the tumor and the motor system < 8 mm [35,36]. This group of patients represented the group of eloquent-motor GBMs and was called group A. The control group (group B) consisted of all the patients with non-eloquent GBMs (defined by a location > 25 mm to the motor system and not infiltrating it) who underwent surgical removal between January 2023 and December 2023. Patients with lesions in language areas were excluded from the study.

**Figure 1:**
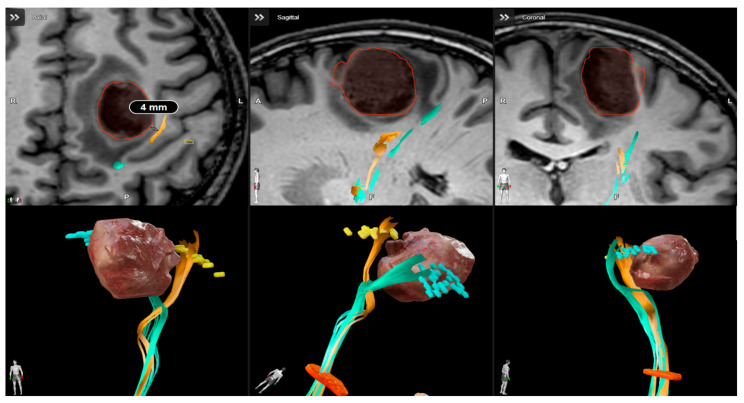
Presurgical planning with Brainlab cranial navigation software for a high-risk motor area glioblastoma. T1 weighted images integrated with nTMS-based DTI reconstruction show the proximity of cortical spinal tract to the lesion.

The diagnosis for both groups was initially supposed with the preoperative MRI and subsequently confirmed by the histologic examination. Patients with glioblastoma relapse, infratentorial glioblastomas or who underwent needle biopsy were excluded from the study. All patients were subjected to neuro-oncological surgery according to the actual standard of care: senior experienced neurosurgeon, intraoperative neuronavigation, and 5-aminolevulinic acid (5-ALA) assisted resection. The protocol in use at our department since March 2020 for motor area lesions is composed by nTMS preoperative motor mapping, nTMS-based CST-tractography reconstruction, intraoperative neurophysiological monitoring (IONM), and mapping with direct cortical stimulation (DCS). All patients of group A were subjected to entire phases of the protocol (Figure 1). The MRI acquisition with a 3T scanner (Ingenia 3T, Philips Healthcare) and the nTMS motor mapping (NBS system 4.3-Nexstim Oy, Elimäenkatu 9 B, Helsinki, Finland) were performed according to our standardized protocol previously described [37]. Stimulation intensity was set individually to determine the resting motor threshold, and motor responses were recorded from upper and lower limb muscles. The functional maps were then integrated into the neuronavigation system and used intraoperatively as a guide for surgical trajectory and cortical entry point. DCS was systematically performed to confirm nTMS findings and refine resection margins. After the postoperative recovery period, all patients underwent multidisciplinary discussion and neuro-oncological evaluation to start adjuvant therapy. Only patients undergoing oncologic therapy according to the Stupp protocol were considered for the study [38].

### 2.2. Data Acquisition

Clinical data were collected within a retrospective revision of our database. We considered age at surgery, sex, tumor location (frontal, temporal, parietal, occipital, or other), histology (comprehensive of molecular issues, IDH, and MGMT-methylation), tumor-track distance (TTD), presurgical tumor volume, postsurgical RV, EOR, complications, KPS index at admission, immediately postsurgical, and at three months of follow-up, motor neurological status expressed by MRC score at surgery, immediately postsurgical, and at three months of follow-up.

#### 2.2.1. Surgical Achievements

The preoperative and postoperative imaging results were reviewed by 2 of the authors in a blind method (L.S. and P.D.Z.) and the tumor volumes were manually measured with the Brainlab planning system. The extent of the resection was retrospectively classified from postoperative MR imaging reports as either GTR or STR by an independent and expert neuroradiologist blind to patient outcome. According to the literature, we considered a GTR in cases of >95% resection and STR in cases of 70–95%. Only patients with postoperative cranial imaging within 48 h of surgery were included in the present study.

#### 2.2.2. Functional Independence

The Karnowski Performance Status (KPS) index was used to classify functional independence, and it was assigned during each clinical visit at admission, at discharge, and at three months after surgery. According to the index, patients with a KPS score ≥ 60 were considered functionally independent, while patients with a KPS score < 60 required considerable medical and daily-life assistance. Similarly, the MRC score was used to check for motor neurological deficit before or after surgery and was assigned during each clinical visit at admission, at discharge, and at three months after surgery. To focus on patients’ functional independence and not just score values, we decided to consider a variation in their clinical status based on their preoperative status. The most effective way to express the centrality of autonomy preservation in patients undergoing surgery seemed to consider KPS and MRC as two dichotomous variables (worsened or improved/modified).

#### 2.2.3. Time to Oncologic Therapy

There are no clear indications in the literature recommending the initiation of cancer therapy within certain terms. However, it is reasonable to think that its delay may adversely affect the patient’s prognosis. The waiting time, expressed in days, for the start of oncologic therapy from the day of surgery was considered for all patients as a parameter that may indirectly affect survival.

#### 2.2.4. Survival

Overall and progression-free survival (OS and PFS) were verified and compared between the two groups during the follow-up period.

### 2.3. Statistical Analysis

The two groups were compared to detect any statistical difference in baseline factors as well as in the parameters achieved after surgery. In addition, analyses were carried out considering any variations in performance status expressed by KPS and MRC or in the waiting time for the start of oncologic therapy. Survival as a function of time after surgery was expressed with the Kaplan–Meier method. The statistical analysis was performed using SPSS software (version 20; SPSS Inc., Chicago, IL, USA). The Pearson chi-square test was used for discrete variables, and the *t*-test for continuous ones. The statistical significance was set at *p* < 0.05.

### 2.4. Patient’s Informed Consent and Ethical Approval

The patients signed specific informed consent forms for MRI acquisition and nTMS examination. The study was conducted in accordance with the ethical standards of the Institutional Research Committee AOUP (Prot. n 0,043,481 27 June 2022) and with the 1964 Declaration of Helsinki, plus later amendments.

## 3. Results

Forty-four patients affected by glioblastoma were enrolled in the study and divided into two groups, consisting of 11 subjects in group A and 33 subjects in group B. For obvious clinical reasons, only patients in group A underwent preoperative study with nTMS motor mapping by two experienced authors (L.S. and S.L.C.). Group A consisted of 5 women (45.5%) and 6 men (55.5%), while group B consisted of 16 women (48.5%) and 17 men (51.5%), all right-handed. At surgery, mean age was 58.4 years (range 45–74 years) in group A and 66.3 years (51–75) in group B, with reasonable significant difference (*p* = 0.015). In group A, the lesion was located in the frontal lobe in 7 cases (63.6%) and in the parietal lobe in 4 (36.4%), while in group B the lesion was located in the frontal lobe in 26 (78.8%) and in the temporal lobe in 7 (21.2%). As expected, cerebral localization demonstrated significant differences between patients with GBM in eloquent and non-eloquent areas (*p* = 0.001). Regarding lateralization, there were no cases of bilateral involvement. The right hemisphere was involved in 5 subjects (45.5%) in group A and 12 (36.4%) in group B; consequently, the left was involved in 6 subjects (54.5%) in group A and 21 (63.6%) in group B (Table 1).

The mean preoperative tumor volume showed a small difference in extension, even if not significant (*p* = 0.123): 18.05 cm^3^ in group A and 26.62 cm^3^ in group B. The median TTD value in group A was 5 mm (1–8). In all cases, nTMS-derived functional boundaries were consistent with intraoperative mapping results. The methylguanine methyltransferase (MGMT) gene was distinguished into methylated and unmethylated. The methylation was found in 7 patients (63.6%) in group A and in 26 patients (81.2%) in group B, while it was not present in 3 patients (27.3%) or 6 patients (18.8%), respectively. In two subjects, one (9.1%) from group A and one (3%) from group B, the data were not available due to the inadequacy of the sample sent for anatomopathological analysis. However, the two groups did not present any significant difference (*p* = 0.449) in terms of MGMT gene status. GBMs are classified according to the expression of the wildtype or mutant isocitrate dehydrogenase (IDH) gene. In both groups, molecular analysis confirmed the presence of the IDH-wildtype gene in all cases.

### 3.1. Surgical Achievements

According to the definition of extension of resection, a GTR had been reached in 8 patients (72.7%) in group A and in 26 patients (78.8%) in group B, while an STR had been reached in the remaining 3 patients (27.3%) in group A and in 7 patients (21.2%) in group B. The statistical analysis did not show a significant difference between the two groups (*p* = 0.678). Complications occurred in two patients (18.2%) in group A, specifically, an ischemic injury due to intraoperative vascular damage and a postoperative epidural hematoma, which was surgically removed without clinical consequences. No complication was noted in group B, with a significant difference of *p* = 0.012.

### 3.2. Functional Independence

**Figure 2:**
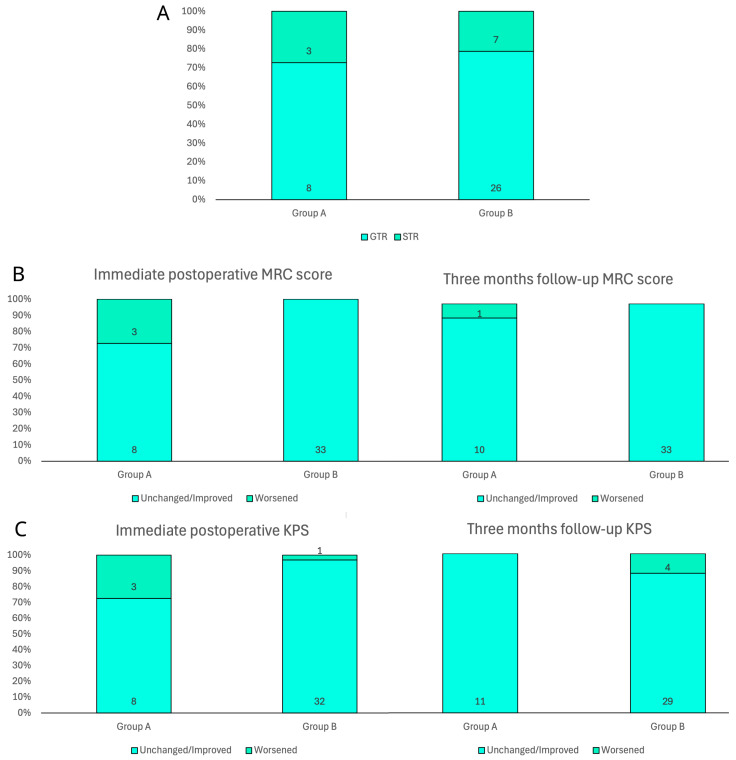
Bar charts of main surgical and clinical outcomes in both groups. (**A**) Extent of resection according to early postoperative MRI evaluation. (**B**) Immediate postoperative and three-month motricity status according to MRC score. (**C**) Immediate postoperative and three-month patient autonomy according to KPS.

Functional independence has been defined by the analysis of motor skills, considering the MRC score, and the daily performance status, according to the KPS index. Both parameters were scheduled in the preoperative and postoperative period, in order to analyze the trend (Figure 2). During the preoperative examination on the day of admission to the department, patients in group A showed a motor deficit (i.e., paresis, hemiparesis) classified as a 3/5 MRC score in four cases (36.4%) and 4/5 in two cases (18.2%). The remaining five patients (45.5%), almost half of the sample, did not show motor deficits. In contrast, in group B, three patients (9.1%) had a preoperative motor deficit with an MRC score of 3/5 and eight patients (24.2%) had 4/5. The remaining 22 patients (66.7%) had complete strength preservation (5/5 MRC score) at preoperative examination. Although apparently not expected, the two groups showed an even distribution of motor impairment (*p* = 0.1), probably due to the extent of peritumoral edema in larger non-eloquent GBMs. On postoperative clinical examination in group A, 3 patients (27.3%) showed an MRC score of 2/5, 1 patient (9.1%) of 3/5, 1 patient (9.1%) of 4/5, and the remaining 6 patients (54.5%) of 5/5, while in group B, no patient showed a severe motor deficit, 4 patients (12.1%) showed an MRC score of 4/5, and the remaining 29 patients (87.9%) of 5/5 (*p* = 0.009). The comparison with the admission clinical report revealed that, in group A, only three patients (27.3%) developed a worsening of their motor status, while the remaining eight patients (72.7%) and the entire group B (100%) maintained or improved their score, with a significant difference of *p* = 0. 002. However, at the three-month follow-up examination, in group A, only 1 patient (9.1%) showed an MRC score of 3/5, 3 patients (27.3%) of 4/5, and the remaining 7 patients (63.6%) of 5/5, while in group B, only 4 patients (12.1%) showed an MRC score of 4/5 and the remaining 29 patients (87.9%) of 5/5 (*p* = 0.091). The comparison with the preoperative motor status revealed that, in group A, only 1 patient (9.1%) developed a worsening, while the remaining 10 patients (90.9%) and the entire group B (100%) maintained or improved their score (*p* = 0. 079).

Regarding the preoperative KPS in group A, 2 patients (18.2%) had a <60 index and the remaining 9 patients (81.8%) had ≥60, while in group B 3 patients (9.1%) had a <60 index and 30 patients (90.9%) had ≥60. Univariate analysis did not show a preoperative significant difference (*p* = 0.411) between the two groups in performance status and demand for assistance in daily living. On postoperative clinical examination of group A, 3 patients (27.3%%) showed a <60 KPS index and 8 patients (72.7%) showed ≥60, while in group B, only 1 patient (3%) showed a <60 KPS index and the remaining 32 patients (97%) showed ≥60 (*p* = 0.015). The comparison with the admission performance status revealed that, in group A, three patients (27.3%) developed a worsening of their KPS index, while the remaining eight patients (72.7%) maintained or improved their score, while in group B only one patient (3%) worsened. The statistical analysis found a significant difference between the two groups (*p* = 0.015). In contrast, at the three-month follow-up examination in group A, all patients (100%) had a ≥60 KPS index, while in group B, 7 patients (21.2%) showed a <60 KPS index and the remaining 26 patients (78.8%) showed ≥60 (*p* = 0.096). The comparison with the preoperative functional status revealed that, in group A, all patients (100%) maintained or improved the score at the three-month follow-up, while in group B, four patients (12.1%) developed a worsening, with a not-significant difference (*p* = 0. 226).

### 3.3. Time to Oncologic Therapy

**Figure 3:**
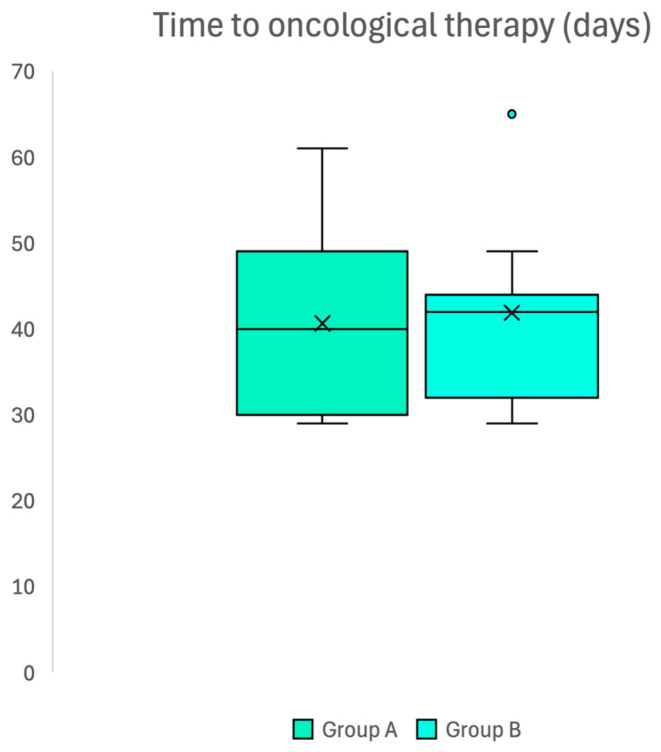
Box plot confirming absence of difference in waiting time to access oncological adjuvant therapy after surgery in two groups.

The mean waiting time for the start of oncologic therapy after surgery was 40.6 ± 11.1 days in group A and 41.9 ± 9.4 days in group B (*p* = 0.719), thus confirming the absence of differences between groups (Figure 3).

### 3.4. Survival

**Figure 4:**
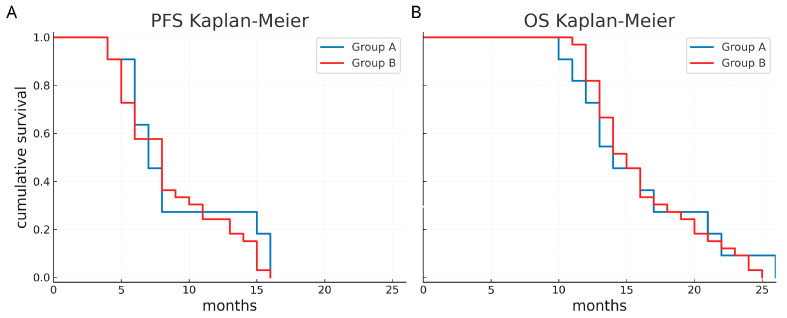
Survival curves according to the two groups. Progression-free survival (**A**) and overall survival (**B**) appear comparable between eloquent and non-eloquent glioblastomas.

The median PFS was 7 months (95% CI 5.4–8.6 months) in group A and 8 months (95% CI 6.4–9.5 months) in group B. The median OS was 14 months (95% CI 10.8–17.2 months) in group A and 15 months in group B (95% CI 13.5–16.5 months). In addition, the 1-year PFS was 27.3% in group A and 24.2% in group B, while the 1-year OS was 72.7% in group A and 81.8% in group B. Kaplan–Meier survival functions did not show a significant difference (*p* = 0.441 and *p* = 0.812, respectively) (Figure 4).

## 4. Discussion

Glioblastoma remains one of the most aggressive and challenging malignancies of the central nervous system, and despite advances in surgical resection and adjuvant therapies, prognosis remains poor [1,2,3,8,9]. In this study, we focused on understanding the impact of glioblastomas located in eloquent areas of the brain, particularly the motor system, and evaluated how our institution’s preoperative planning protocol, incorporating nTMS motor mapping, affects surgical outcomes and survival. Our results show that EOR and the presence of postoperative neurological deficits continue to be crucial determinants of survival in GBM patients, irrespective of tumor location. This aligns with previous studies that have emphasized the importance of achieving maximal safe resection to improve OS and PFS in GBM patients [38,39,40]. Interestingly, in this cohort, both groups achieved a relatively high rate of gross total resection and OS did not significantly differ between eloquent and non-eloquent GBMs. This contrasts with the assumption that eloquence inherently worsens prognosis and suggests that, despite the challenging nature of motor area tumors, our protocol, which includes nTMS motor mapping, allows for aggressive resection without compromising surgical safety, in agreement with recent studies that have demonstrated the utility of nTMS in glioma surgery [19,21,22,30].

The use of nTMS motor mapping has been widely validated in recent years as a tool to improve the precision of glioma resection in the motor cortex. Several studies have shown that preoperative nTMS mapping can predict eloquent cortical areas with high accuracy, leading to a reduction in postoperative neurological deficits [19,41,42]. This aligns with the findings of our study, where the incorporation of nTMS into the preoperative protocol was pivotal in planning resections for glioblastomas located in motor areas. Additionally, the ability of nTMS to provide a functional map of the motor cortex before surgery allows for more informed intraoperative decisions, thereby enhancing surgical outcomes [19,22,30,37,43]. However, the analysis of functional independence postoperatively indicated a trend toward increased neurological deficits in the eloquent motor area group (group A), with 27.3% of patients showing worsened motor status at discharge, compared to the non-eloquent group. This finding emphasizes the inherent risk of surgery in eloquent brain areas and supports the hypothesis that even a minimal decrease in motor function could severely impact patients’ quality of life and functional status. Interestingly, at three months, the two groups showed a similar recovery in motor function, with the majority of patients either maintaining or improving their motor score, underscoring the potential for recovery, especially when appropriate neurosurgical techniques and rehabilitation are employed [44]. This means that the proposed protocol was able to limit postoperative deficits to a degree of reversibility that did not have a long-term impact. While nTMS provides an accurate preoperative functional map that guides the surgical strategy, IONM remains the intraoperative gold standard for the real-time monitoring of motor pathways. The two techniques are not alternatives but rather synergistic: nTMS enhances preoperative planning and risk stratification, whereas IONM allows continuous intraoperative feedback, thus increasing surgical safety. The combination of both modalities likely represents the most reliable approach to maximize the surgical outcome [41,42].

The time to initiate adjuvant oncological therapy did not significantly differ between groups (*p* = 0.719), which could indicate that both sets of patients underwent similar multidisciplinary treatment regimens. While the timing of adjuvant therapy may not have had a significant impact on OS or PFS in this cohort, previous studies have suggested that the early initiation of therapy might influence long-term outcomes [16,17,19]. The delay in starting oncological therapy could be partly due to the postoperative recovery and rehabilitation time, which may vary based on the extent of the motor deficits and the functional status of patients after surgery.

Regarding survival, our analysis showed no significant difference in median OS (14 months in group A versus 15 months in group B; *p* = 0.812) or PFS (7 months in group A versus 8 months in group B; *p* = 0.441). These results suggest that, despite the higher risk of functional deficits in motor area glioblastomas, the oncological prognosis does not significantly differ when compared to non-eloquent glioblastomas, when advanced surgical techniques and prompt adjuvant therapies are applied. This finding is consistent with the literature which indicates that, although the location of the tumor in eloquent areas may increase surgical risks, it does not necessarily correlate with a worse oncological prognosis [39].

This study has several limitations that must be acknowledged. First, the small cohort size inevitably reduces the statistical power influencing the conclusions. In this regard, our results should be considered as preliminary but valuable stimuli for larger multicenter studies. Second, the age imbalance between groups represents a potential confounder. This difference may in part reflect earlier diagnoses in motor-eloquent tumors leading to surgery at a younger age. Similarly, the slightly smaller tumor volumes in eloquent cases may be related to this earlier presentation. Although neither variable showed a significant impact in our series, both may have influenced outcomes. Taken together, these limitations underscore that our results should be interpreted with caution, yet they also highlight the clinical importance of the question addressed. Despite the inherent weaknesses of a small, retrospective cohort, our findings suggest that a comprehensive preoperative protocol including nTMS motor mapping, CST tractography, and IONM may mitigate the oncological impact of eloquence and warrant confirmation in larger prospective studies.

## 5. Conclusions

Motor-area GBMs pose a significant challenge due to their high surgical risk. Despite the risks of postoperative neurological deficits, survival outcomes appear comparable between eloquent and non-eloquent glioblastomas when treated with a structured preoperative protocol incorporating nTMS motor mapping, which enables safe and aggressive resections without compromising oncologic outcomes. The integration of these techniques may mitigate the oncological impact of eloquence in glioblastoma prognosis, improving patient management and survival. However, further studies are needed to clarify the long-term potential role of this protocol in improving both functional and oncological outcomes in this challenging patient population.

## Figures and Tables

**Table 1:** Summary of the main features of the cohorts of patients with glioblastoma in eloquent (group A) and non-eloquent area (group B).

Population Characteristics	Group A (*n* = 11)	Group B (*n* = 33)	*p* Value
Mean age, years (min–max)	58.4 (45–74)	66.3 (51–75)	0.015
Gender, n (%)			
Female	5 (45.5)	16 (48.5)	0.862
Male	6 (54.5)	17 (1.5)	
Handedness, n (%)			
Right	11 (100)	33 (100)	-
Left	0 (0)	0 (0)	
Tumor location, n (%)			
Frontal	7 (63.6)	26 (78.8)	
Parietal	4 (36.4)	0 (0)	0.001
Temporal	0 (0)	7 (21.2)	
Hemisphere, n (%)			
Right	5 (45.5)	12 (36.4)	0.592
Left	6 (54.5)	21 (63.6)	
Preoperative tumor volume (cm^3^)	18.05	26.62	0.123

## Data Availability

The original contributions presented in this study are included in the article. Further enquiries can be directed to the corresponding author.

## Abbreviations

The following abbreviations are used in this manuscript:

nTMS	Navigated transcranial magnetic stimulation
IONM	Intraoperative neurophysiological monitoring
DCS	Direct cortical stimulation
WHO	World health organization
CST	Cortical spinal tract
PFS	Progression-free survival
OS	Overall survival
